# ID 195 - Uso de Linfócitos T Específicos no Tratamento da Infecção por Citomegalovírus em Receptores de Transplante de Células Hematopoiéticas: Uma Revisão Sistemática

**DOI:** 10.5327/2237-9622.2025.v34s1.195

**Published:** 2025-11-25

**Authors:** Tayna Felicissimo Gomes de Souza Bandeira, Luciana Cavalheiro Marti, Edna Terezinha Rother, Clarissa Martins Machado

**Keywords:** citomegalovírus, transplante de medula óssea, linfócitos T específicos

## Abstract

**Introdução:**

O citomegalovírus (CMV) representa uma ameaça significativa após o transplante de células hematopoiéticas (TCH). As estratégias de controle incluem a profilaxia com letermovir ou a terapia preemptiva com ganciclovir (PET). Sem profilaxia, 65% a 90% dos receptores soropositivos desenvolvem uma infecção por CMV clinicamente significativa. Devido às desvantagens da PET, a profilaxia com letermovir é preferível, pois reduz eventos relacionados ao CMV e melhora a sobrevida geral. No entanto, o CMV-CS refratário ou resistente continua sendo um desafio, com maribavir apresentando eficácia limitada.

**Método:**

Esta revisão sistemática seguiu o Manual Cochrane e as diretrizes PRISMA (), registrada no PROSPERO (CRD42023440361). As buscas foram feitas nas bases PubMed, Scopus, Embase e Web of Science em 19 de junho de 2023, atualizadas em 17 de julho de 2024, incluindo dois artigos adicionais sem restrições de língua ou ano. Os critérios de inclusão foram pacientes de TCH com CMV-CS refratário ou inelegíveis a terapias convencionais; imunoterapia com células T específicas para hCMV; e resultados como taxa de resposta, , eventos adversos e morte por CMV. Os critérios de exclusão incluíram uso profilático, pacientes não refratários, resumos de congressos e revisões narrativas.

**Resultados:**

Dos 1.895 registros identificados, 614 duplicatas foram removidas, e 1.153 estudos foram excluídos. Onze estudos (2012-2024) incluíram 255 receptores de TCH recebendo imunoterapia adotiva (IA), principalmente com células T específicas para CMV. A doença enxerto-versus-hospedeiro (GvHD) ocorreu em 1,82% dos casos, e eventos adversos em 4,4%. A síndrome de liberação de citocinas (CRS) leve foi observada em 1,3% dos pacientes. A eficácia, avaliada em 299 pacientes, mostrou uma taxa média de resposta de 78,2%. A recorrência do CMV-CS foi de 24,4% em 213 pacientes, e a morte por CMV foi relatada em 9,7% de 307 pacientes.

**Conclusão:**

Com a chegada do letermovir, um antiviral seguro para profilaxia de CMV, melhorou-se o manejo de CMV-CS em TCH. No entanto, falhas na profilaxia podem levar a episódios recorrentes e resistência antiviral. O letermovir substituiu temporariamente a terapia adotiva na profilaxia, mas não no tratamento de CMV-CS refratário. Esta revisão sistemática incluiu nove estudos sobre a segurança e eficácia da imunoterapia adotiva em receptores de TCH com CMV-CS refratário. Os estudos eram observacionais e apresentaram viés. A DNAemia é um biomarcador importante, devendo seguir definições rigorosas. A taxa de resposta à imunoterapia foi de 76%, superior à do maribavir (57%). A revisão mostrou que a recorrência de CMV foi de 24% após AI, e a mortalidade por CMV em tratamentos refratários pode ultrapassar 30%.

